# Measuring molecular interactions in solution using multi-wavelength analytical ultracentrifugation: combining spectral analysis with hydrodynamics

**DOI:** 10.1042/bio04102014

**Published:** 2019-04-01

**Authors:** Borries Demeler

**Affiliations:** University of Lethbridge and the Canadian Center for Hydrodynamics in Lethbridge, Canada, and the University of Montana, USA

## Abstract

In 1926, the Swedish scientist Theodor Svedberg was awarded the Nobel Prize in Chemistry for his work on a disperse system, and for studying the colloidal properties of proteins. This work was, to a large extent, made possible by his invention of a revolutionary tool, the analytical ultracentrifuge. These days, technological advances in hardware and computing have transformed the field of analytical ultracentrifugation (AUC) by enabling entirely new classes of experiments and modes of measurement unimaginable by Svedberg, making AUC once again an indispensable tool for modern biomedical research. In this article these advances and their impact on studies of interacting molecules will be discussed, with particular emphasis on a new method termed multi-wavelength analytical ultracentrifugation (MWL-AUC). Novel detectors allow us to add a second dimension to the separation of disperse and heterogeneous systems: in addition to the traditional hydrodynamic separation of colloidal mixtures, it is now possible to identify the sedimenting molecules by their spectral absorbance properties. The potential for this advance is significant for the study of a large range of systems. A further advance has occurred in data management and computational capabilities, opening doors to improved analysis methods, as well as direct networking with the instrument, facilitating data acquisition and data handling, and significant increases in data density from faster detectors with higher resolution capability.

## A brief history

AUC has been around for nearly a century. Thanks to the introduction in 1947 of the first commercially produced instrument, the Spinco Model E, AUC quickly became widespread in biochemistry and life science research laboratories. Its main application was the characterization of biopolymers to elucidate molecular composition and molar mass. In the 1970s, AUC fell by the wayside when cheaper and easier alternatives emerged, such as gel electrophoresis, which could be used to accomplish the same task, while the Model E was difficult and time-consuming to use and expensive to maintain. This calculus changed in the early 1990s when Beckman introduced the Proteomelab XLA, a fully digitized instrument featuring absorbance optics. Later, Rayleigh interference optics were added (termed the XLI), as well as a fluorescence detector sold by Aviv Biomedical. In 2006, the first MWL detector for AUC (MWL-AUC) was developed by H. Cölfen and colleagues. Several improvements followed over the years, and in 2017 Beckman Coulter offered a new commercial instrument, the Optima AUC, capable of MWL-AUC detection. With this instrument, crucial technological advances were realized with greatly enhanced temporal, radial and spectral resolution.

## The basics

**Figure 1:**
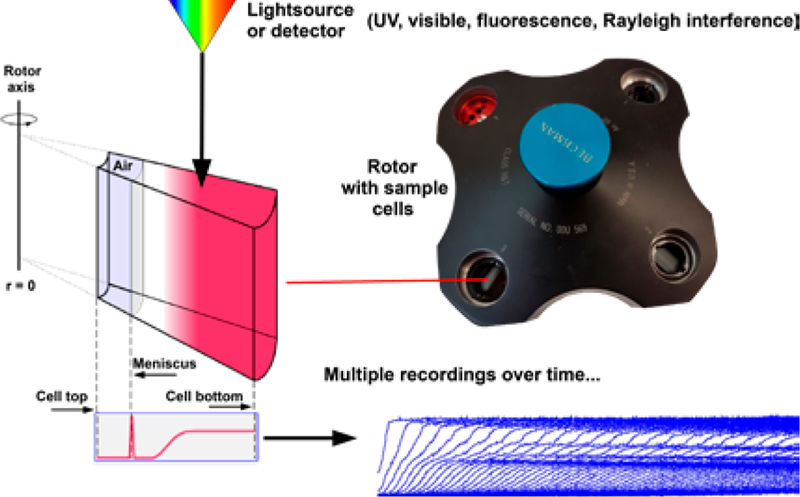
Schematic of an AUC experiment. A light source illuminates a sedimenting and diffusing solution of molecules in a sector-shaped compartment while the rotor is spinning. Different optical systems collect data representing concentration distributions of the sample as a function of time and radius.

AUC is a biophysical technique that uses centrifugal force to separate molecules dissolved in solution based on their size, anisotropy and buoyant density. Figure 1 shows a schematic overview of an AUC experiment. Commercially available instruments support the detection of molecules by UV-visible absorbance, Rayleigh interference and fluorescence emission. The rotor speed can be varied up to 60,000 revolutions per minute, generating nearly 300,000 g at the bottom of the centrifuge cell. AUC is a first-principle technique that does not require any standards. The primary variables determined from AUC experiments are the sedimentation, and the diffusion coefficients and the partial concentrations of any molecules present in a mixture of solutes. These parameters can be used to obtain additional information about other molecular properties (see Box 1 for details).

The experimental sedimentation and diffusion coefficients are determined by fitting an optical signal trace, obtained over the radial range of the sample compartment at multiple time points (see Figure 1), to finite element solutions of the Lamm equation, a partial differential equation that describes the sedimentation and diffusional transport in an AUC cell. Due to the complexity of the fitting calculation these computations are typically performed on supercomputers to maximize speed and resolution of the analysis.

## New tools for measuring molecular interactions

With the arrival of MWL detection, AUC has a new trick to offer: in addition to the traditional hydrodynamic separation of different solutes in a mixture, unique chromophores present in different solutes in the mixture can now be exploited to distinguish these molecules by detecting their distinct spectral contributions to the absorbance patterns measured for the mixture. This opens interesting avenues for analysis: interacting molecules in solution typically exist in multiple states, which can be either free in solution or complexed. Molecules that form complexes can exist in multiple stoichiometries. Determining these stoichiometries has traditionally been very challenging using hydrodynamic characterization alone, since mass/concentration resolution is either not sufficiently accurate, or dynamically changing reaction boundaries prevent the determination of discrete hydrodynamic states. Reaction boundaries represent compositions that gradually change with concentration, and hence reflect mass-action dynamics proportional to the local concentration environment in the sedimenting boundary, and proportional to the kinetics of the reaction observed in the non-equilibrium transport process. Since reacting components in a mixture are rarely examined at concentrations at either end of the binding isotherm, it is common to see mixtures of reactants and products with changing composition along the gradient. This prevents the unique assignment of molar masses of the sedimenting and diffusing species. To complicate matters further, molar masses can only be determined if accurate partial specific volumes can be assigned to a sedimenting species. However, the partial specific volume changes as a function of association stoichiometry for dissimilar interacting molecules such as proteins and nucleic acids, lipids and carbohydrates. MWL-AUC offers an exciting new tool to solve this problem.

## How does MWL analysis work?

Molecules display unique absorbance spectra based on their chemical composition and atomic arrangements, as well as the solvents in which they are dissolved. When two molecules interact to form a complex, two different absorbance spectra observed for each of the two pure molecules combine to form a new absorbance spectrum in a mostly additive manner. There are some exceptions: a hypo- or hyperchromic effect is seen when rearrangements of the microenvironment of atoms upon binding cause a change in the observed spectrum, but these changes are typically small and can often be neglected. For additional detail please refer to Box 2.

**Figure 3:**
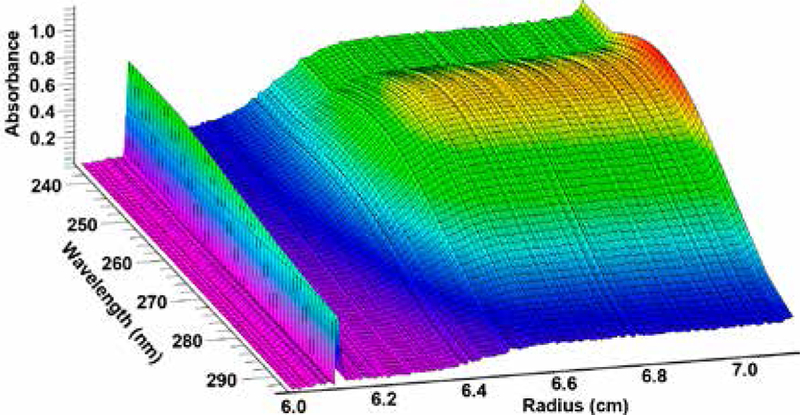
A single scan from a 3D multi-wavelength experiment. Each wavelength mapped on the y-axis represents a separate boundary typically observed in a traditional single-wavelength experiment. Each radial position, mapped on the x-axis, provides a complete wavelength scan of the species sedimenting at that radial position. At approximately 6.1 cm the meniscus of the solution column is visible. The absorbance of this surface is mapped to the z-axis.

In a sedimentation velocity experiment, molecules present in mixtures are separated based on their size, density and frictional properties. This means that at each point in the sedimenting boundary, a different composition may be present, and hence a different spectrum will be observed. Decomposition of the observed spectrum into its basis spectra can be accomplished by solving Equation 5 with non-negatively constrained least squares analysis. It recovers the relative amount of each component at each radial position in the boundary. Integrating the contributions from each basis spectrum to each hydrodynamically separated species over the entire experiment provides the stoichiometry of the hydrodynamically separate species’ composition. A single scan of a MWL sedimentation velocity is shown in Figure 3. It is important to note that the stoichiometry alone does not necessarily reveal information regarding the correct oligomeric size, and that the hydrodynamic information does not necessarily inform about the correct stoichiometry, since alone both methods provide degenerate answers. For example, a 1:2 stoichiometry could reflect both a complex of *AB*_*2*_ or *A*_*2*_*B*_*4*_. Likewise, without knowledge of the precise stoichiometry, the precise partial specific volume, which is a weight-average of the individual partial specific volumes of each constituent species, is not known, and the molar mass obtained from the sedimentation velocity experiment using Equation 1 is subject to error. Thus, adding the spectral information to the hydrodynamic information has the distinct advantage of providing a *unique* solution for both stoichiometry and molar mass. The linear decomposition of Equation 5 results in amplitudes for each spectral species *S*_*i*_, one amplitude for each radial position at each time point (radial scan) of the experiment. Together, these amplitudes represent a traditional sedimentation velocity experiment, one for each spectrally distinct species used for the decomposition. Here, it is important that all spectral contributors, even absorbing buffer components, are included in the decomposition to assure a good fit in the decomposition of Equation 5. The UltraScan software incorporates routines for conveniently processing MWL-AUC data on supercomputers. After decomposition, the resulting 2D datasets are analysed just like traditional single-wavelength experiments with methods implemented in UltraScan. The only difference is that the hydrodynamic information from each 2D dataset only reflects a single chemically distinct species, and any complexes it is forming. Hydrodynamic species that are identical in multiple 2D datasets represent complexes formed. If the intrinsic absorbance spectra *S*_*i*_ are expressed in molar concentration, then the relative concentration of each species in the complex directly yields the stoichiometry of the association. Furthermore, the molar mass derived with the mass-averaged partial specific volume can then be used to ascertain the oligomeric state consistent with the resulting stoichiometry. Molar concentrations of free and complexed species obtained in this fashion can then be used directly to calculate equilibrium constants for these reactions.

## Applications of MWL-AUC

MWL-AUC experiments offer a powerful new way to add spectral information content to traditional hydrodynamic separation by AUC. Systems where hetero-interactions between two different molecules are investigated can benefit from MWL-AUC, as long as the hetero-associating partners have sufficiently different absorbance properties. The spectral differences may be subtle, but as long as a sufficiently large number of wavelengths over the distinct range are analysed, the analysis will be able to resolve the individual components. For more information on resolving multiple spectra please review the information in Box 3. Given the large number of wavelengths that can be analysed with either the Cölfen or the Optima AUC MWL optics, even 15°–20° are often sufficient to obtain good spectral separation. Typical interactions between biopolymers such as protein–DNA or protein–RNA interactions are extremely well suited for MWL analysis because of the complementarity of their spectra between 220–300 nm, a wavelength range very well suited for AUC experiments. At 220 nm, the peptide bond in the backbone of a protein is a strong absorber while nucleic acids have relatively little absorbance, conversely, at 260 nm nucleic acids absorb strongly, while aromatic amino acid side chains in proteins only contribute weakly in this region. Another class of proteins include those that have strong absorbance bands in the visible regions, such as certain metalloproteins and proteins containing haem groups. Even differences in the absorbance ratio of aromatic side chains and the total number of peptide bonds can be exploited when measuring interactions between unlabelled proteins. To further enhance spectral differences, artificial labels such as an Alexa fluor, or fusions with fluorescent proteins can be added, which now exist in many different colours, to add unique spectral absorbance properties to the system under investigation.

## Summary

In addition to the traditional hydrodynamic separation of macromolecular mixtures, MWL-AUC offers spectral identification of these separated molecules for molecules that have unique absorbance spectra. This adds important detail to the macromolecular characterization and facilitates the measurement of heterogeneous interactions. Simultaneous spectral and hydrodynamic characterization enhances the ability to identify stoichiometries and molar masses of heterocomplexes. The advances brought about by MWL-AUC provide a compelling new tool for studying interactions of molecules in the solution phase, and enhance an old, but enduring and already essential technique for studying complex biological systems.

## Figures and Tables

**Figure 2:**
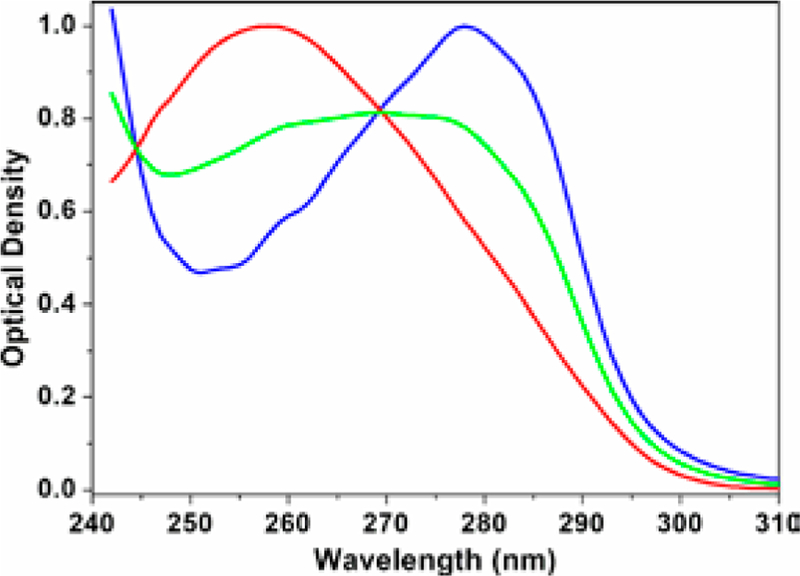
Mixtures of protein and DNA. When DNA (red) and protein (blue) are mixed, the spectra of each molecule combine in an additive fashion to produce a new spectrum for the mixture (green).

## Box 1

The Svedberg equation provides the molar mass *M* of a solute:

Equation 1. $$M=\frac{RTs}{D(1-\bar{\nu }\rho )}$$

where *R* is the universal gas constant, *T* is the temperature in Kelvin, *s* and *D* represent the sedimentation- and diffusion coefficients, $\bar{\nu }$ is the partial specific volume of the molecule, and ρ is the density of the solvent. Both sedimentation and diffusion are inversely proportional to the friction of the molecule:

Equation 2. $$s=\frac{M(1-\bar{\nu }\rho )}{Nf}\;\;D=\frac{RT}{Nf}$$

where *f* is the frictional coefficient and *N* is Avogadro’s number. Using the Stokes–Einstein relationship we can use the frictional coefficient to derive the hydrodynamic radius, *R*_*h*_, of the molecule:

Equation 3. $$R_{h}=\frac{f}{6\pi \eta }$$

where *η* is the viscosity of the solvent. The hydrodynamic radius represents the radius of an assumed sphere that has the same frictional coefficient as the sedimenting and diffusing molecule, which may not be spherical at all. If the partial specific volume of the molecule is known, the anisotropy (level of non-globularity) of the molecule can also be obtained by calculating the frictional ratio, which is the ratio of the actual frictional coefficient, *f*, over the frictional coefficient, *f*_*0*_, of a hypothetical sphere that has the same volume as the molecule of interest. *f*_*0*_ can be obtained by deriving the volume of the molecule, and employing the Stokes–Einstein relationship again:

Equation 4. $$V=M\frac{\bar{\nu }}{N}\;\;r_{0}={\left(\frac{3V}{4\text{π}}\right)}^{1/3}\;\;f_{0}=6\text{πη}r_{0}$$

## Box 2

Mathematically, the spectrum of the complex can be represented as a linear combination of the basis spectra *S*_*i*_ from any number, *n*, of pure molecules:

Equation 5. $$S_{complex}=\sum_{i=1}^{n}c_{i}S_{i}$$

where *S*_*i*_ is the observed spectrum for the complex, and *c*_*i*_ is the partial contribution of the *i*^*th*^ component’s basis spectrum. If the spectra *S*_*i*_ are known and sufficiently different, any mixture can now be decomposed into the precise amount each component contributes to the spectrum of the complex, or mixture of components (see Figure 2). From this, the stoichiometric contributions can be obtained.

## Box 3

A convenient metric for the difference between two spectra *u* and *v* is the angle θ between them:

Equation 6. $$\theta =\arccos\left[\frac{u\cdot v}{\left\|u\right\|\cdot \left\|v\right\|}\right]$$

An angle of 0° means that the two spectra are linearly dependent, or simply multiples of each other, while an angle of 90° degrees indicates perfect orthogonality. A larger angle provides better spectral separation. A larger angle can often be achieved by including a larger range of wavelengths in the experiment.
